# Real-time sensing of war’s effects on wellbeing with smartphones and smartwatches

**DOI:** 10.1038/s43856-023-00284-y

**Published:** 2023-04-17

**Authors:** Merav Mofaz, Matan Yechezkel, Haim Einat, Noga Kronfeld-Schor, Dan Yamin, Erez Shmueli

**Affiliations:** 1 Department of Industrial Engineering, Tel-Aviv University, Tel-Aviv, Israel; 2 School of Behavioral Sciences, The Academic College of Tel Aviv-Yafo, Tel-Aviv, Israel; 3 School of Zoology and Sagol School of Neuroscience, Tel-Aviv University, Tel-Aviv, Israel; 4 Center for Combating Pandemics, Tel-Aviv University, Tel-Aviv, Israel; 5 MIT Media Lab, Cambridge, MA USA

**Keywords:** Public health, Biomarkers

## Abstract

**Background:**

Modern wars have a catastrophic effect on the wellbeing of civilians. However, the nature of this effect remains unclear, with most insights gleaned from subjective, retrospective studies.

**Methods:**

We prospectively monitored 954 Israelis (>40 years) from two weeks before the May 2021 Israel-Gaza war until four weeks after the ceasefire using smartwatches and a dedicated mobile application with daily questionnaires on wellbeing. This war severely affected civilians on both sides, where over 4300 rockets and missiles were launched towards Israeli cities, and 1500 aerial, land, and sea strikes were launched towards 16,500 targets in the Gaza Strip.

**Results:**

We identify considerable changes in all the examined wellbeing indicators during missile attacks and throughout the war, including spikes in heart rate levels, excessive screen-on time, and a reduction in sleep duration and quality. These changes, however, fade shortly after the war, with all affected measures returning to baseline in nearly all the participants. Greater changes are observed in individuals living closer to the battlefield, women, and younger individuals.

**Conclusions:**

The demonstrated ability to monitor objective and subjective wellbeing indicators during crises in real-time is pivotal for the early detection of and prompt assistance to populations in need.

## Plain language summary

This study investigated the impact of the May 2021 Israel-Gaza war on the wellbeing of Israeli civilians. To do so, 954 Israelis over the age of 40 were monitored for six weeks before and after the war using smartwatches and a mobile application that asked daily wellbeing questions. The researchers found that during the war, people experienced spikes in heart rate, decreased sleep quality and duration, and increased screen time. These changes were more significant in people living closer to the battlefield, women, and younger individuals. However, after the ceasefire, wellbeing indicators returned to baseline levels. The study shows that monitoring wellbeing in real-time during crises can help identify and assist populations in need.

## Abstract (web summary)

Mofaz et al. prospectively monitor older Israeli participants, from two weeks before the May 2021 Israel-Gaza war until four weeks after the ceasefire, using smartwatches and wellbeing questionnaires in a mobile app. They identify changes in wellbeing indicators during missile attacks and throughout the war, that fade in most after it is over.

## Introduction

The 20th century was the most war-ridden century recorded in history—and the one most fatal for civilians^1^. Indeed, the last decades are marked not only by advances in the arms industry and changes in warfare strategies but also by the shift of the battlefield into civilian areas, making them more vulnerable in modern wars. This shift is reflected in the stark increase in war-related civilian fatalities, from 5% of casualties at the turn of the 19th century to 15% during World War I (WW I), 65% by the end of World War II, and more than 90% in wars that erupted during the 1990s^2^.

The immense number of civilian casualties is but one aspect of the devastating consequences for civilians. Another is the masses of civilians suffering from war-related invisible psychological scars^3^. Indeed, wars were found to have severe, long-lasting effects on mental health and wellbeing^4^. These effects include, among others, reduced happiness, anxiety, fear, depression, and post-traumatic stress disorder (PTSD)^5–7^. Markedly, a recent study suggested that one in five people living in conflict-affected regions in the previous ten years will experience depression, anxiety, PTSD, bipolar disorder, or schizophrenia^8^. The recent Russian invasion of Ukraine was already reported to have extraordinary mental impacts on Ukrainians and vulnerable people worldwide and is likely to cause a large-scale mental health crisis in the near future^9^. In light of this circumstance, healthcare systems and relief organizations must conduct targeted investigations and prepare for the provision of mental health support to vulnerable populations immediately^9^.

The wide, easy access to media, in general, and to unencumbered social media, in particular, was shown to further increase individuals’ fear levels^10^ and preoccupation with disturbing thoughts regarding mortality^11^. For example, after the 2015 terror attack in France, media consumption was associated with insomnia^12^. Moreover, by its nature, media has the capacity to extend the effect far beyond those directly influenced by the conflict.

Despite the valuable knowledge gleaned from the above studies, the vast majority of them are based on questionnaires that were administered retrospectively. Given that memory loss and delayed recall are widely documented during traumatic events, there is a serious drawback in assessments that rely on retrospective analysis^13^. Moreover, self-reports rely on the individual’s perception, whose relation to the actual effect on well-being remains to be studied.

Smartphones and smartwatches can serve as a valuable tool for assessing a population’s well-being during crises as they enable the objective, continuous monitoring of a variety of measures. For example, smartphones and smartwatches have been recently employed by researchers to investigate the effects of lockdowns on wellbeing^14^. Several recent studies further showed that wearable sensors could be even more sensitive than humans in detecting physiological changes following infection with and vaccination against COVID-19^15,16^. In the context of mental health, their ability to continuously record objective measures such as heart rate, heart rate variability, and sleep patterns make them adequate for monitoring early markers of PTSD^17,18^.

Here, we used objective and subjective, continuous, rich data from smartphones and smartwatches worn by Israeli citizens in order to examine changes in various well-being indicators before, during, and after the May 2021 armed conflict between Israel and Gaza. The May 2021 Israel-Gaza conflict severely affected civilians on both sides. Over 4300 rockets and missiles were launched toward Israeli cities, and 1500 aerial, land, and sea strikes were launched towards 16,500 targets in the Gaza Strip^19^. We conducted a prospective observational study where 954 participants received smartwatches and downloaded a dedicated mobile application we developed for their smartphones. The smartwatches continuously monitor various measures, including heart rate, step count, sleep start hour, and sleep modes. The smartphones also collected sensory data on screen-on time, activity modes, and GPS location. The mobile application included a daily questionnaire with questions relating to mood, stress, sleep duration and quality, physical activity, and social encounters.

We identify considerable changes in all the examined well-being indicators during missile attacks and throughout the war, including spikes in heart rate levels, excessive screen-on time, and a reduction in sleep duration and quality. These changes, however, faded shortly after the war, with all affected measures returning to baseline in nearly all the participants. Greater changes are observed in individuals living closer to the battlefield, women, and younger individuals. The ability to monitor objective and subjective well-being indicators during crises in real-time is pivotal for the early detection of and prompt assistance to populations in need.

## Methods

### Study design

We analyze data collected as part of the PerMed study^14,20^ to ascertain the effects of the May 2021 Gaza-Israel war on various indicators of well-being in the population. The study was approved on August 8, 2021, by Tel-Aviv University’s Institutional Review Board (IRB) and was conducted under strict protocol guidelines.

Participants in the PerMed study were a convenience sample recruited by a professional survey company, via advertisements on social media. During the time frame used for analysis in the current study, recruitment of participants was limited to older adults, aged 40 or more. The participants were equipped with a Garmin^®^ Vivosmart 4 smartwatch and installed a dedicated mobile application that we developed (PerMed App).

We define four time periods: (1) Baseline period (B)—the 2 weeks before the war, i.e., April 26–May 9, 2021; (2) War period (W)—May 10–20, 2021; (3) First “back to routine” period (R1)—the first and second weeks after the war, i.e., May 21–June 3, 2021; and (4) Second “back to routine” period (R2)—the 3rd and 4th weeks after the war, i.e., June 4–17, 2021.

### Inclusion criteria

We included the study participants of the PerMed study who were above the age of 40 and were active in the PerMed study throughout the evaluation period (i.e., they joined the PerMed study before April 26, 2021, and remained in the study at least until June 17, 2021).

### Participant recruitment and engagement

In order to recruit subjects and keep them engaged throughout the PerMed study, we hired a professional survey company. The survey company used advertisements on social media for recruitment of individuals from the general population. The survey company was responsible for guaranteeing that participants met the study’s requirements, including their willingness to fill an app questionnaire three times a week and wear a smartwatch during the entire study. Eligible participants received a detailed explanation of the study, after which they were requested to sign a digital consent form. Then, participants were asked to fill a one-time enrollment questionnaire and to install two apps on their smartphones: the Garmin Connect app, which was used to collect data from their smartwatch, and the dedicated PerMed app, which we developed to collect GPS-based location and to allow participants to fill the daily questionnaires.

To improve the quality and reliability of the data and to ensure its continuous collection, we applied the following measures. First, participants who did not fill the daily questionnaire by 7 p.m. received a notification in their mobile app to fill the questionnaire. Second, we developed a dedicated dashboard. The dashboard, which was monitored regularly, helped us identify data collection issues, such as participants who did not fill the questionnaires at least three times a week or participants who did not wear their smartwatches. Such participants were contacted by the survey company, and were encouraged to cooperate better. The dashboard also helped us to identify issues that were not related to participants’ cooperation, such as bugs in the mobile app. This identification allowed us to respond faster and provide timely solutions. Supplementary Fig. 1 further illustrates the quality of the data used in the current study.

### Data collection

We used the following sources of data:

- Self-reported indicators collected through the Permed App’s daily questionnaire: reported mood level (on a scale of −2 to 2, where −2 means awful and 2 means excellent); reported stress level (on a scale of −2 to 2, where −2 means very low and 2 means very high); reported sleep duration (in hours), reported sleep quality (on a scale of −2 to 2, where −2 means awful and 2 means excellent), reported sport time (in minutes), and the reported number of social encounters. A detailed description of the questionnaire is provided in the Supplementary Methods.
- Daily aggregated indicators collected through the Garmin smartwatch: step count, average heart rate (bpm), sleep start hour, and percentage of awake time during night sleep (seconds).
- Daily aggregated indicators collected by the smartphone sensors (only applicable for Android-based smartphones): Screen-on time (in hours), which is known to be highly correlated with stress^21^, and the percentage of time still (according to Google Activity Recognition).

The 12 indicators were then grouped into three main categories: (1) Mental-related indicators: screen-on time, reported mood level, reported stress level, and the reported number of social encounters; (2) Energy expenditure-related indicators: step count, average heart rate, percentage of time still, and reported sport time; and (3) Sleep-related indicators: awake time, sleep start hour, reported sleep duration, and reported sleep quality.

In addition to these 12 indicators, we also collected:

- Information from the enrollment questionnaire, which included questions about age, gender, income level, and city of residence.
- Location data from the smartphone: location data (GPS coordinates) was sampled every 15 min and was used to determine the area where each participant resides (see subsection “Data preprocessing”).

### Data preprocessing

Before analyzing the data, we performed several preprocessing steps. First, if participants filled the daily questionnaire more than once on the same day, only the latest questionnaire for that day was considered. The rationale behind this decision was that a questionnaire, once filled, was sent to the server, and could not be updated anymore. Therefore, in the case of a filling error, participants were instructed to re-fill the questionnaire.

Then, for each participant and for each of the 12 indicators, we calculated a single weighted average value for each of the four periods—baseline (B), war (W), first “back to routine” (R1), and second “back to routine” (R2). The weighted average for each participant was calculated by first averaging the corresponding well-being indicator values separately for work days and free days. Then, the weighted average of these two values was calculated by giving a weight of 5/7 to work days and 2/7 to free days. The rationale for calculating this weighted average is further demonstrated in Supplementary Fig. 2. In brief, we identified a weekly rhythm across various indicators, where free days (weekends and national holidays) exhibited different mean daily values than work days. Since the examined time periods were relatively short, and some included more free days than others (e.g., holidays), we wanted to correct for a potential bias.

To differentiate the direct effects of the missile attacks from indirect ones, such as empathy with relatives and friends, we also examined the correlation between the participants’ area of residence and its distance from Gaza to the 12 well-being indicators. To determine the most up-to-date area of residence for each participant, we examined all the GPS coordinates collected for each participant around 04:00 a.m. during the baseline period and selected the most frequent ones. When participants did not have GPS data during the baseline period nights, we used the hometown stated in the enrollment questionnaire. Then, we divided the participants into three exposure groups based on the proximity of their area of residence to Gaza: (1) high exposure—less than 60 km from Gaza, (2) medium exposure—between 60 and 110 km from Gaza, (3) low exposure—more than 110 km from Gaza. These 60 and 110 km thresholds were selected based on both the distance from Gaza (the number of missiles launched towards areas closer to Gaza was considerably higher) and the effective time to reach a shelter once an air-raid siren is sound^22^. More details regarding the classification into exposure groups are provided in Supplementary Note 1 and Supplementary Fig. 3.

### Statistics and reproducibility

First, we assessed the short-term changes in the heart rate of participants following sirens. To do so, for each participant and for each siren that was sound in their area of residence, we compared the baseline heart rate before the siren and the affected heart rate following the siren. The baseline value was defined as the average heart rate during the time frame of 45 min to 15 min before the siren started. The affected value following a siren was calculated as the maximum heart rate during the time frame of 5 min before and 15 min after the siren. Finally, we calculated the difference between those two values for each participant and siren, and the average of those differences for each participant. We considered only sirens that were sound between 2 a.m. and 6 a.m. to minimize the effects of other factors, such as physical activity, on the heart rate. Moreover, sirens for which we identified another siren during the baseline time frame were excluded from the analysis, in order to isolate the effect of the examined siren.

In order to test the longer-term effect of the war on different well-being indicators, we used a separate Mixed ANOVA test for each of the above-noted 12 indicators; in each test, the considered indicator served as the dependent variable. For the independent variables (main factors), the within-subjects factor was the time period, comprised of four levels: baseline, war, first “back to routine,” and second “back to routine”. The between-subjects factor was the distance-based exposure group, comprised of three exposure levels: high, medium, and low. More formally, for each of the 12 well-being indicators, the considered Mixed ANOVA model includes the two main factors and their interaction:

1 $${{{{{\rm{Indicator}}}}}}\,\sim	\, {{{{{\rm{Time}}}}}}\_{{{{{\rm{Period}}}}}}+{{{{{\rm{Exposure}}}}}}\_{{{{{\rm{Group}}}}}}\\ 	+\, {{{{{\rm{Time}}}}}}\_{{{{{\rm{Period}}}}}}\,\ast\, {{{{{\rm{Exposure}}}}}}\_{{{{{\rm{Group}}}}}}$$

To better understand the characteristics of individuals who were more affected by the war, we performed a second (post hoc) ANOVA analysis. For this analysis, we analyzed the data of individuals in the exposure risk groups that were found to be affected by the war in our previous Mixed ANOVA analysis. More specifically, for each of the 12 indicators noted above, we used a separate ANOVA test. For each test, the dependent variable was defined as the difference between the corresponding indicator values during the war and the baseline periods. For the independent variables, we considered: (1) Exposure group—a factor with two levels: medium and high (the two affected exposure risk groups according to the previous analysis). (2) Age group—a factor with two levels: Younger (59 and below) and Older (60 and above), where the groups were divided based on the median age; (3) Gender—a factor with two levels: Men and Women; (4) Income Level— a factor with three levels: Below median, median, and above the median. We also adjusted for the effect of the following independent variable: (5) Baseline level—a factor with two levels: Below the population median value and equal to or greater than the population median value (before the war). More formally, for each of the 12 well-being indicators, the considered ANOVA model can be described by the following equation:

2 $$\Delta {{{{{\rm{Indicator}}}}}}\,\sim	\, {{{{{\rm{Exposure}}}}}}\_{{{{{\rm{Group}}}}}}+{{{{{\rm{Age}}}}}}\_{{{{{\rm{Group}}}}}}+{{{{{\rm{Gender}}}}}}\\ 	+ {{{{{\rm{Income}}}}}}\_{{{{{\rm{Level}}}}}}+{{{{{\rm{Baseline}}}}}}\_{{{{{\rm{Level}}}}}}$$

It should be noted that each ANOVA test was performed over the subset of participants who had at least one value in free days and at least one value in work days for the corresponding indicator, during all considered time periods (four periods for the first Mixed ANOVA analysis, and two periods for the second analysis). In other words, the number of participants considered in each test may vary.

Post hoc analyses for ANOVA tests’ significant interactions were performed using Bonferroni post hoc tests, and effect sizes were measured by Cohen’s d^23^.

All statistical analyses were performed using IBM SPSS Statistics version 27 and Python 3.7.

### Reporting summary

Further information on research design is available in the Nature Portfolio Reporting Summary linked to this article.

## Results

### Descriptive statistics

The study included a cohort of 954 participants above the age of 40 with a median age of 59. Of the 954 participants, 549 (57.55%) were women and 405 (42.45%) were men. The reported income of 475 (49.79%) participants was above the median income level, while that of 185 (19.39%) was in the median income range, and that of 258 (27.04%) was below the median; 36 did not answer the relevant question in the enrollment questionnaire. In terms of exposure to missile attacks, 68 (7.13%) participants lived in high-risk areas, 704 (73.79%) in medium-risk areas, and 182 (19.08%) were not exposed to missile attacks at all. Table 1 provides further information about the participants’ characteristics. As presented in detail in Supplementary Fig. 1a, participants exhibited a high level of cooperation with the study requirements.

**Table 1:** Characteristics of the study participants.

Characteristic	All participants (*N* = 954)	High risk (*N* = 68)	Medium risk (*N* = 704)	Low risk (*N* = 182)
**Age**				
40–49	9.33% (89)	20.59% (14)	7.1% (50)	13.74% (25)
50–59	42.66% (407)	47.06% (32)	40.62% (286)	48.9% (89)
60–69	32.6% (311)	22.06% (15)	35.37% (249)	25.82% (47)
≥70	15.41% (147)	10.29% (7)	16.9% (119)	11.54% (21)
**Gender**				
Men	42.45% (405)	47.06% (32)	40.62% (286)	47.8% (87)
Women	57.55% (549)	52.94% (36)	59.38% (418)	52.2% (95)
Income^*^				
Above median	49.79% (475)	63.24% (43)	49.43% (348)	46.15% (84)
Median	19.39% (185)	22.06% (15)	18.89% (133)	20.33% (37)
Below median	27.04% (258)	13.24% (9)	27.98% (197)	28.57% (52)
Unspecified	3.77% (36)	1.47% (1)	3.69% (26)	4.95% (9)

*The median income specified in the questionnaire was 15,000 NIS per household.

### Changes in heart rate following a siren

**Fig. 1:**
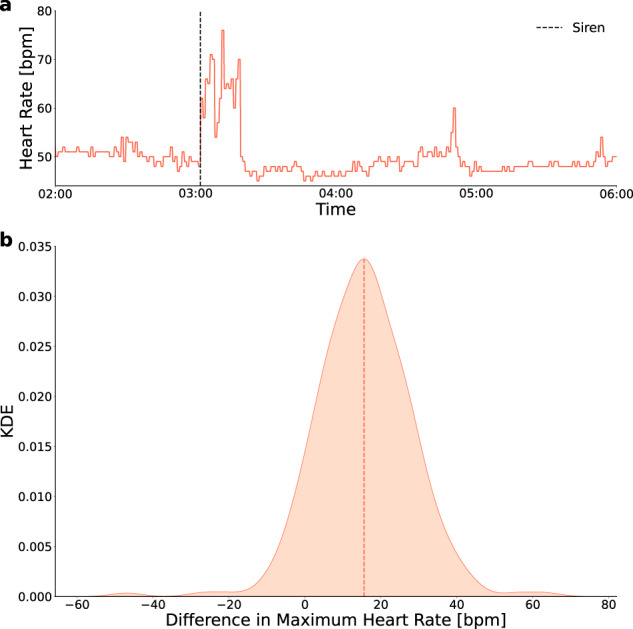
Changes in heart rate following a siren. **a** An example of the change in heart rate for a single participant following a siren. The x-axis represents the time and the y-axis is the heart rate of the participant in beats per minute. Dashed, black, vertical line—the time period in which the siren was heard. **b** Distribution of the changes in heart rate following a siren compared with the baseline value over all participants. Since each participant could experience several sirens, each participant is represented by the mean of his/her changes. The x-axis represents the mean change in heart rate value and the y-axis is the kernel density estimator (KDE). Dashed, orange, vertical line—median value of the distribution.

During the war, when incoming missiles were identified by defense systems, sirens were raised as a means to prompt civilians to head to shelters. In Fig. 1, we demonstrate the changes in the heart rate of participants following these sirens. Specifically, Fig. 1a demonstrates the change in heart rate of a single participant—before, during, and after a siren. As can be seen from the figure, the siren led to a considerable increase in the heart rate of that individual—from an average baseline value of roughly 50 bpm in the hour before the siren to a maximum value of 76 bpm in the hour after the siren. The heart rate returned to baseline values after about 20 min. Figure 1b shows the distribution of changes in heart rate following the raising of sirens compared to baseline values over all eligible participants. For each participant, the baseline value was defined as the average heart rate during the time frame of 45 to 15 min before the siren started. The value following siren initiation was calculated as the maximum heart rate during the time frame of 5 min before and 15 min after the siren. The median value of the distribution (15.4 bpm) indicates that the heart rate of most of the participants increased considerably in response to the siren.

### Effects of the war on various wellbeing indicators

Table 2 presents the *p* values of the within-subjects effects for the Mixed ANOVA test that was conducted for each of the 12 examined well-being indicators. Each row represents a single indicator (i.e., a single test). A table detailing the mean values and the number of participants included in each test is available in Supplementary Table 1. Post hoc test results are presented only for pairs of time periods that include the baseline period.

**Table 2:** Within-subjects effects of the Mixed ANOVA tests.

		Time period factor				Time period * exposure group interaction			
		Main ANOVA	Post hoc			Main ANOVA	Post hoc		
			B & W	B & R1	B & R2		B & W	B & R1	B & R2
Mental	Screen-on time [hour]	0.00***	0.00*** (↑)	1	1	0.00***	Low exposure: 0.003** (↑) Medium exposure: 0.00*** (↑) High exposure: 0.00*** (↑)	Low exposure: 1 Medium exposure: 0.736 High exposure: 1	Low exposure: 1 Medium exposure: 1 High exposure: 1
	Reported mood level	0.00***	0.00*** (↓)	0.08	0.47	0.00***	Low exposure: 0.133 Medium exposure: 0.00*** (↑) High exposure: 0.00*** (↑)	Low exposure: 0.242 Medium exposure: 0.237 High exposure: 1	Low exposure: 1 Medium exposure: 0.152 High exposure: 1
	Reported stress level	0.00***	0.00*** (↑)	1	1	0.00***	Low exposure: 0.26 Medium exposure: 0.00*** (↑) High exposure: 0.00*** (↑)	Low exposure: 1 Medium exposure: 1 High exposure: 1	Low exposure: 1 Medium exposure: 0.916 High exposure: 1
	Reported number of encounters	0.00***	0.007**(↓)	1	0.571	0.642	-	-	-
Energy	Step count	0.00***	0.00*** (↓)	0.374	1	0.140	-	-	-
	Average heart rate [bpm]	0.002**	0.00*** (↓)	1	1	0.488	-	-	-
	% time still	0.00***	0.00***(↑)	1	1	0.476	-	-	-
	Reported sport time [min]	0.00***	0.00*** (↓)	0.195	0.684	0.003**	Low exposure: 0.627 Medium exposure: 0.00*** (↓) High exposure: 0.00*** (↓)	Low exposure: 1 Medium exposure: 1 High exposure: 0.426	Low exposure: 0.362 Medium exposure: 0.657 High exposure:1
Sleep	Awake time [sec]	0.00***	0.00*** (↑)	1	1	0.245	-	-	-
	Sleep start hour	0.003**	0.021* (↑)	0.396	1	0.495	-	-	-
	Reported sleep duration [hour]	0.004**	0.008** (↓)	1	0.624	0.021*	Low exposure: 1 Medium exposure: 0.00*** (↓) High exposure: 0.013* (↓)	Low exposure: 1 Medium exposure: 1 High exposure: 1	Low exposure: 1 Medium exposure: 1 High exposure: 0.781
	Reported sleep quality	0.00***	0.00*** (↓)	1	1	0.00***	Low exposure: 1 Medium exposure: 0.00*** (↓) High exposure: 0.00*** (↓)	Low exposure: 1 Medium exposure: 1 High exposure: 1	Low exposure: 1 Medium exposure: 1 High exposure: 0.771

Each row represents a mixed ANOVA test for a single well-being indicator. The two “Main ANOVA” columns represent the *p* value obtained by the mixed ANOVA test for the time period factor and time period * exposure group interaction. The other six columns present the *p* values obtained by Bonferroni post hoc tests for different pairs of time periods (applied only for significant “main ANOVA” effects), where B indicates the baseline period, W the war period, R1 the first “back to routine” period, and R2 the second “back to routine” period.

Statistically significant effects are marked with stars and the direction of these effects is marked with corresponding arrows. A table detailing the mean values and the number of participants included in each test is available in Supplementary Table 1.

***p  <  0.001, **p  <  0.01, *p  <  0.05.

**Fig. 2:**
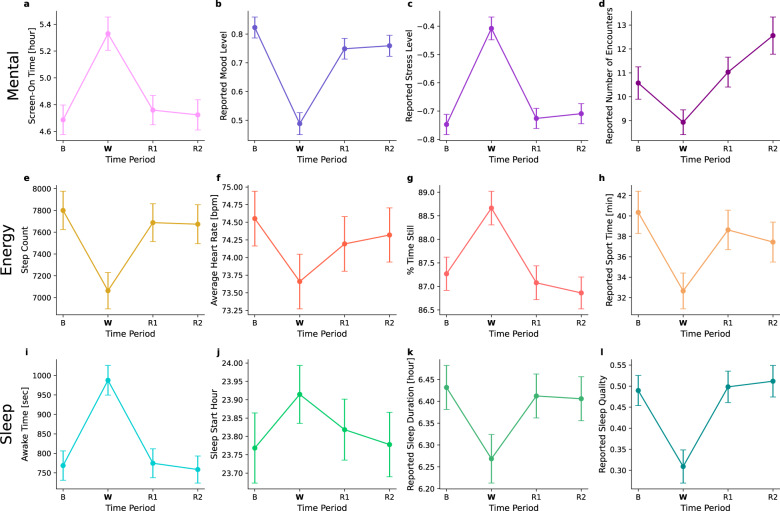
Effects of the war on the entire population for various wellbeing indicators. The presented well-being indicators include **a** Screen-on time in hours, **b** Reported mood level, **c** Reported stress level, **d** Reported number of encounters, **e** Step count, **f** Average heart rate in beats per minute, **g** Percentage of time still, **h** Reported sport time in minutes, **i** Awake time during night sleep in seconds, **j** Sleep start hour, **k** Reported sleep duration in hours, and **l** Reported sleep quality. The x-axis represents four time periods: baseline period (B), war period (W), first “back to routine” period (R1), and second “back to routine” period (R2). The y-axis represents the mean value for the examined well-being indicator. Error bars represent a single standard error.

Overall, we found that: (1) the war significantly affected the general population with respect to all the examined well-being indicators (Table 2 and Fig. 2); (2) the effects were significantly larger in areas with a higher risk of exposure to missile attacks; and (3) the changes diminished almost entirely within the 2-week period after the war.

More specifically, we observed that among the mental-related indicators, the screen-on time increased by 38.601 min during the war (*p* < 0.001, Cohen’s d: 0.28), the reported mood level decreased by 0.33 (*p* < 0.001, Cohen’s d: 0.52), the reported stress level increased by 0.34 (*p* < 0.001, Cohen’s d: 0.52), and the reported number of encounters decreased by 1.64 encounters (*p* < 0.01, Cohen’s d: 0.16). As to the energy expenditure-related indicators, during the war, there was a decrease of 736.9 steps in the step count (*p* < 0.001, Cohen’s d: 0.19), a decrease of 0.89 bpm in the average heart rate (*p* < 0.001, Cohen’s d: 0.11), an increase of 1.4% in the percentage of time still (*p* < 0.001, Cohen’s d: 0.2), and a decrease of 7.69 min in the reported sport time (*p* < 0.001, Cohen’s d: 0.23). With regard to the sleep-related indicators, during the war, the awake time increased by 219 s during night sleep (*p* < 0.001, Cohen’s d: 0.32), the sleep start hour was delayed by 8.76 min (*p* < 0.05, Cohen’s d: 0.09), the reported sleep duration decreased by 9.78 min (*p* < 0.01, Cohen’s d: 0.18), and the reported sleep quality decreased by 0.18 (*p* < 0.001, Cohen’s d: 0.28). It should be noted that as opposed to Fig. 1, which demonstrates a sharp though relatively brief increase in heart rate following the sirens, the decrease in average heart rate in Fig. 2f is aggregated over the entire war period. The latter is consistent with the general decrease in energy expenditure indicators. Namely, during the war period, individuals tended to reduce their activity and this may have caused their average heart rate levels to decline as well.

Similarly to the acute and clear effects of the war, the data also indicate a quick recovery immediately after the war ended. Specifically, we observed an immediate return to baseline values in all 12 indicators within the first 2 weeks after the war (R1) (Table 2, Fig. 2 and Supplementary Table 1). With the exception of the reported number of encounters (which continued to rise), this return to baseline values remained stable also in the succeeding 2-week period (R2). This remarkable recovery is also evidenced by the data presented in Supplementary Notes 2 and Supplementary Fig. 4, showing that the changes between the baseline period (B) and the first “back to routine” period (R1) are distributed roughly normally around 0, with a relatively small standard deviation and seemingly symmetric tails. While the general population demonstrated a clear resilience, we also identified a few individuals who did not return to their baseline levels (Supplementary Notes 2, Supplementary Fig. 4, Supplementary Notes 3, Supplementary Fig. 5).

**Fig. 3:**
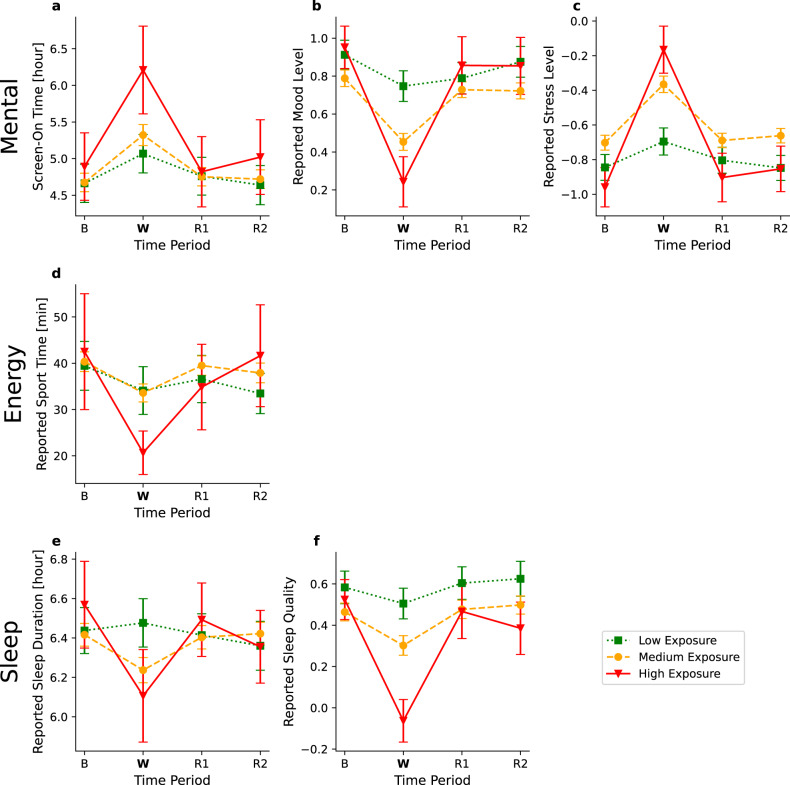
Effects of the war by exposure group for various well-being indicators. The presented well-being indicators include **a** Screen-on time in hours **b** Reported mood level, **c** Reported stress level, **d** Reported sport time in minutes, **e** Reported sleep duration in hours, and **f** Reported sleep quality. The x-axis represents four time periods: baseline period (B), war period (W), first “back to routine” period (R1), and second “back to routine” period (R2). The y-axis represents the mean value for the examined well-being indicator. Error bars represent a single standard error. The three plots represent the exposure groups: high exposure (solid red), medium exposure (dashed orange), and low exposure (dotted green).

In terms of area of residence, a measurably stronger effect was observed in individuals living in areas with a higher risk of exposure to missile attacks (Table 2, Fig. 3 and Supplementary Table 1). More specifically, a significant interaction was found between the time period and exposure group factors in six of the 12 examined indicators: screen-on time (*p* < 0.001), reported mood level (*p* < 0.001), reported stress level (*p* < 0.001), reported sport time (*p* < 0.01), reported sleep duration (*p* < 0.05) and reported sleep quality (*p* < 0.001). These significant interactions suggest that the war had a different effect on the three exposure groups. Post hoc analyses indicate a significant increase from baseline in the screen-on during the war period in all exposure groups (high: *p* < 0.001, Cohen’s d: 0.48; medium: *p* < 0.001, Cohen’s d: 0.28, low: *p* < 0.01, Cohen’s d: 0.17). For the other indicators, the change between the baseline and war period was significant only for the high and medium exposure groups. Specifically, we observed a decrease in the reported mood level (high exposure group: *p* < 0.001, Cohen’s d: 1.21; medium exposure group: *p* < 0.001, Cohen’s d: 0.51), an increase in the reported stress level (high exposure group: *p* < 0.001, Cohen’s d:1.33; medium exposure group: *p* < 0.001, Cohen’s d: 0.5), a decrease in the reported sport time (high exposure group: *p* < 0.001, Cohen’s d: 0.48; medium exposure group: *p* < 0.001, Cohen’s d: 0.22), a decrease in the reported sleep duration (high exposure group: *p* < 0.05, Cohen’s d: 0.42; medium exposure group: *p* < 0.001, Cohen’s d: 0.2), and a decrease in the reported sleep quality (high exposure group: *p* < 0.001, Cohen’s d: 1.23; medium exposure group: *p* < 0.001, Cohen’s d: 0.24). Moreover, the effect on the high-exposure group was considerably stronger than that experienced by the medium-exposure group in all six indicators.

### Subgroups post hoc analysis

To better understand the characteristics of individuals who were more affected by the war, we performed a second (post hoc) ANOVA analysis. For this analysis, we analyzed the data of individuals in the exposure risk groups that were found to be affected by the war in Table 2 (i.e., Medium and High), and tested the association between exposure risk group, age group, gender, and income level, to the observed effect of the war, adjusting for the baseline levels of individuals. We found a stronger effect in the high-risk exposure group than in the medium-risk exposure group in five out of the six indicators that were found to be significant in Table 2 (screen-on time: *p* < 0.01, reported mood level: *p* < 0.01, reported stress level: *p* < 0.01, reported sport time: *p* < 0.01, and reported sleep quality: *p* < 0.001), even when controlling for the additional factors. A stronger effect was found in women than in men in terms of screen-on time (*p* < 0.05, mean difference value of 49.32 min for women vs. 29.53 for men) and reported stress level (*p* < 0.01, mean difference value of 0.47 for women vs. 0.3 for men). Additionally, participants in the younger age group experienced a greater decline in heart rate (*p* < 0.05, mean difference value of −1.32 bpm for the younger group vs. −0.66 for the older group). Tables detailing the *p* values, the mean difference values, and the number of participants considered in each ANOVA test are available in Supplementary Tables 2, 3.

## Discussion

The current study evaluated the real-time effects of the May 2021 Israel-Gaza war on the well-being of the older Israeli population (40 years of age and above). To the best of our knowledge, this is the first study to use a combination of objective (smartphone and smartwatch sensors) and subjective (self-reported questionnaires) measures in real-time, before, during, and after a war.

The main findings of our study are that Israelis showed an acute and robust reaction to the war situation. The reaction was demonstrated in sharp responses in both objective and subjective measures related to three axes, mental (screen-on time, mood level, stress level, and the number of social encounters), energy expenditure (step count, average heart rate, percentage of time still, and sport time), and sleep (awake time during night sleep, sleep start hour, sleep duration, and sleep quality) (Fig. 2). All these measures worsened during the war. Interestingly, our data suggest the resilience (the ability of the individual to “bounce back” after the war^24^) of the Israeli civilian population: as quick as the effects of the war were, so was the recovery, with all the measures that were altered during the war period returning approximately back to normal within 2 weeks after the cease-fire date. We also identified larger effects in individuals who lived closer to the battlefield, women, and younger individuals.

Studies of war’s effects on civilians are not rare. A recent meta-analysis that explored the prevalence of depression and PTSD among civilians after wars in Africa, Asia, and the Balkans (two and more years after the end of the war) found their rate to be ~27 and 26%, respectively^25^. These percentages vary greatly across studies, with smaller studies usually showing higher rates of psychopathology^25^. These results may appear to contrast with our data showing a fast recovery from war-induced changes in well-being indicators. These differences may stem from a number of factors related to individual as well as environmental and social aspects. One clear difference between the various Israel-Gaza armed conflicts in the last decades and other wars is that, at least on the Israeli side, these are short (days to weeks) wars defined by a very specific threat (missiles targeting civilian centers). Moreover, much of the population have means to protect themselves against this threat, including warning sirens, safe rooms or shelters built to withstand missiles, and the Iron Dome missile-defense system. Additionally, these wars are abruptly terminated with some type of cease-fire that usually holds for a few years. Not to undermine the significant experience of Israeli citizens, it could be argued that this is an untypical type of war and, therefore, the stress experienced differs from those described in most of the other studies concerning war. Interestingly, a study that examined post-war PTSD symptoms in Israeli citizens following the war between Israel and Hezbollah (July-August 2008) showed that symptoms appeared only in 7.2% of the population compared to the much higher rates discussed above^26^.

An additional factor that should be taken into consideration is the repeated exposure of adults in Israel to war situations. The Israeli population is exposed to missile attacks every few years (in areas close to the border with Gaza, the attacks have been continuous over the last decade), as well as repeated acts of terrorism throughout the country. Such chronic exposure can either result in resilience or produce a long-term negative effect^27,28^. In some cases, the effects of repeated stress were reported to have worse consequences compared with those due to acute stress. For example, a study of US military personnel serving in Iraq and Afghanistan during the last two decades showed that the number of psychiatric diagnoses and psycho-social problems is higher in the second and third deployments compared with the first deployment, and it may also be associated with the total length of time spent in deployment, i.e., the exposure to chronic and repeated stress^29^. However, other studies, including a large body of more biologically oriented research, suggest that at least under some conditions, exposure to repeated stress results in the habituation of responses. For example, human subjects who repeated a stressful task exhibited attenuated cardiovascular reactivity and improved task performance^30^. A recent study in rats demonstrated that exposure to repeated restrain stress results in a clear habituation of both the HPA (hypothalamus-pituitary-adrenal axis, one of the main biological response pathways for stress) and cardiovascular response^31^. Such studies have led to the idea of “stress inoculation”, i.e., that exposure to measured stress may result in higher resilience toward later stresses^32^. An in-depth investigation of these possibilities should be a target of future work.

Another factor that could influence our results is the age of the participants. In most previous studies, younger adults were found to be less resilient than middle-aged or older adults^28,33–37^. Our study focused on older adults, aged 40 or more. This observation could be explained by repeated exposure with age. For example, a 60-year-old Israeli resident would have been directly exposed over the course of his or her lifetime to four or five major wars (depending on the definition of war) and to numerous limited armed conflicts. Therefore, it is possible that the results will be different when studying younger populations. Moreover, because participants in this study were a convenience sample, they may not necessarily represent the Israeli population of ages 40 and above.

It would be of interest to conduct an equivalent study to our on the inhabitants of the Gaza Strip. A study of children and adolescents in Gaza following the 2012 war indicated that ~30% of the children who were exposed to the war developed PTSD as well as other disorders^38^. A broader meta-analysis of studies regarding the Palestinian population in the areas under the jurisdiction of the Palestinian Authority and in the Gaza Strip showed a high rate of PTSD and anxiety disorders in the population. While the repeated hostilities affect both Israelis and Palestinians in the Gaza Strip, it is possible that each group’s responses may differ. The differences can be explained at many levels.

Second, beyond war, the people of Gaza are exposed to many more stresses compared to the Israeli population, including poverty, insufficient health care and education, minimal prospects at the individual level, an authoritative regime, and more^39,40^. Accordingly, it is not far-fetched to suggest that under such horrid conditions, the outbreak of war can trigger the appearance of a severe psychiatric disorder. However, we were unable to locate much data about Gaza in the existing literature. Therefore, as it could be of importance to use high-level data to compare the Israeli and Gaza Strip populations, we would like to call upon scientists from the Gaza Strip to contact us for the purpose of conducting collaborative work aimed at examining people from both sides of the border, for the mutual benefit of all.

Another interesting result is the significant contribution of the proximity of the area of residence to Gaza to the effects of the war. We found that individuals residing in areas with high exposure to missile attacks (less than 60 km from Gaza) had significantly stronger effects than individuals with medium exposure (between 60 and 110 km from Gaza), while individuals residing in areas with low or no exposure (more than 110 km from Gaza, i.e., out of the Gaza missile range) showed no significant response to the war other than an increase in screen-on time. These differences make sense as areas closer to Gaze were characterized by a considerably higher number of missiles launched towards them and a considerably shorter time to reach a shelter once an air-raid siren was sounded. Nevertheless, such differences were not found in a previous study that compared individuals living in southern Israel, who are subject to frequent missile attacks from Gaza, to a group living in Northern Israel, out of the missile range^41^. However, in that study, the researchers used questionnaires administered four months after the end of the war, which may explain the different results. In addition, future research should assess the conflict intensity’s direct impact (e.g., the number of missiles launched towards an area), aside from its indirect impact through exposure risk groups, on civilians’ reactions.

Although at the population level, our and previous studies suggest that most individuals are resilient, it is important to note that some people are not, and therefore are at risk of suffering from long-term effects^42,43^. Therefore, early identification of at-risk sub-populations and individuals is crucial. In this study, we were able to identify subgroups of individuals who were more affected by the war—individuals who lived closer to the battlefield, women, and younger individuals. This insight could aid decision-makers in providing assistance in a more efficient manner. To test the differences between subgroups, we used a simple ANOVA test. In principle, a panel regression analysis would be more appropriate in this case. However, since, on average, participants filled the daily questionnaires only two to three times a week, such analysis would be of limited value. Future work should aim at collecting more frequent data from participants to enable panel regression analysis, as it could add valuable insights into the complex relationships between variables over time.

We also tested the possibility of using our approach for the early detection of non-resilient individuals and studied the capacity of the individual to recover from the war (individual resilience). Indeed, we were able to identify individuals who showed similar changes as the entire population in the transition from pre-war to war, but did not show recovery during the four weeks after the war. For example, Supplementary Notes 3 and Supplementary Fig. 5 presents the case of a participant who exhibited increased stress, reduced mood, reduced activity (step count and reported sport time), shorter sleep duration, and reduced sleep quality during the war compared to pre-war measures, and all these measures remained altered for the four weeks after the war. It is possible that changes in these measures, such as reduced mood, increased stress, reduced activity, reduced interactions, and disturbed sleep, especially when persistent for over 4 weeks, may be related to the development of psychopathology, including PTSD and major depression^44,45^. Our data were not obtained using clinically validated scales or diagnoses. Future research should examine whether individuals who do not show recovery based on well-being indicators similar to those used in this study, are indeed at elevated risk of developing psychopathology. Such individuals could be flagged for referral to a mental health professional.

Another limitation of the study is the use of one-item self-reported measures whose reliability and validity was put into question by various studies. Clearly, using more elaborate and complex tools may result in more detailed and reliable measures. However, when attempting to measure a high number of dimensions with high frequency, as done in this study, it is important to keep the questions simple and their number as low as possible.

Any real-world system implementing our approach must consider the following two aspects. First, our approach may raise ethical concerns due to the sensitivity of the data being collected and analyzed. Any real-world implementation must explicitly declare the purpose for which the data were collected, and take all necessary precautions to prevent data misuse. Second, it would be desired to minimize the use of subjective measures to the minimum required (e.g., in order to simplify the interaction with the users). Specifically, despite the rich information they provide on a wide set of well-being dimensions, some subjective measures can be replaced or be partly induced from one or more other subjective and/or objective measures. For example, as detailed in Supplementary Notes 4 and Supplementary Fig. 6, self-reported mood and self-reported stress are highly correlated. Likewise, reported sport time was found to be highly correlated with the smartwatch’s step count.

To sum, we presented an approach for real-time sensing of war’s effects on well-being with smartphones and smartwatches. We demonstrated how this approach could offer insights into changes in well-being indicators during and after a war, and allow the identification of populations in need. Given the continuing global impact of wars, in general, and the ongoing Russian invasion of Ukraine, in particular, such identification becomes crucial. In principle, this study sets the know-how for simple, effective, and automatic monitoring of populations not only during war situations but also during other crises or scenarios requiring special attention.

## Supplementary information

Supplementary Information

Reporting Summary

## Data Availability

Data required to reproduce the results presented in this paper are available in the GitHub repository^46^: https://github.com/permedtau/war_effects.

## Code availability

The source code is available in the GitHub repository^46^: https://github.com/permedtau/war_effects.
