# Supplementary material for: Real-time sensing of war’s effects on wellbeing with smartphones and smartwatches
Source: Commun Med (Lond). 2023 Apr 17;3:55. doi: 10.1038/s43856-023-00284-y (PMC10109229; doi:10.1038/s43856-023-00284-y)
Supplement: Supplementary file 1 — Supplementary Information [file 43856_2023_284_MOESM1_ESM.pdf]

Supplementary Information

Real-time sensing of war's effects on wellbeing with smartphones and smartwatches

Merav Mofaz[1], Matan Yechezkel[1], Haim Einat[2], Noga Kronfeld-Schor[3], Dan Yamin[1,4,+], Erez Shmueli[1,5,+,*]

[1] Department of Industrial Engineering, Tel-Aviv University, Tel-Aviv, Israel
[2] School of Behavioral Sciences, The Academic College of Tel Aviv-Yafo, Tel-Aviv, Israel
[3] School of Zoology and Sagol School of Neuroscience, Tel-Aviv University, Tel-Aviv, Israel
[4] Center for Combating Pandemics, Tel-Aviv University, Tel-Aviv, Israel
[5] MIT Media Lab, Cambridge MA, USA
[+] These authors contributed equally
[*] Corresponding author: shmueli@tau.ac.il

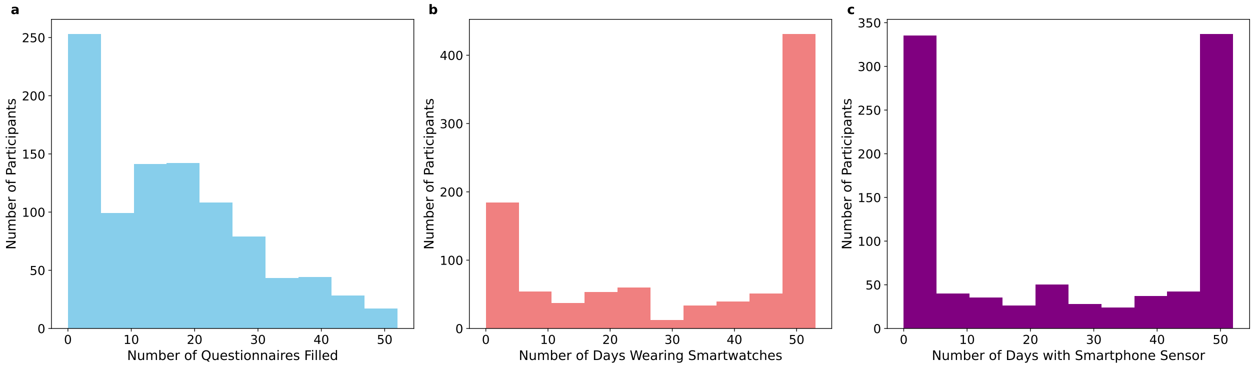

Supplementary Figure 1. Data quality: (a) Number of questionnaires filled during the 53 days of the study, (b) Number of days smartwatches were worn during the 53 days of the study (c) Number of days with smartphone sensor data. Supplementary Figure 1a shows the distribution of the number of questionnaires filled by the participants during the 53 days of the study. The average number of days with questionnaire answers was 16.14 out of 53 days (30.19%). Supplementary Figure 1b shows the distribution of the number of days smartwatches were worn during the study. The average number of days with smartwatch data was 33.01 and the median was 43 out of 53 days (81.13%). Supplementary Figure 1c shows the distribution of the number of days data from the smartphone sensors were collected. The average number of days with smartphone sensor data was 25.97 and the median was 25 out of 53 days (47.17%).

Supplementary Methods

The daily questionnaire included the following eight questions:

1. How is your mood today?
   - Awful (-2)
   - Bad (-1)
   - OK (0)
   - Good (1)
   - Excellent (2)
2. How many hours of sleep did you have last night?
3. How would you define your last night sleep quality?
   - Awful (-2)
   - Bad (-1)
   - OK (0)
   - Good (1)
   - Excellent (2)
4. Try to remember how many minutes of sports activity you performed on the last day?
5. How would you describe the level of your stress during the last day?
   - Very Low (-2)
   - Low (-1)
   - Medium (0)
   - High (1)
   - Very high (2)
6. Try to estimate the number of people you have been with in the last day for up to 2 meters away?
7. Have you been diagnosed by a qualified medical professional with any of the following diseases? I was not diagnosed by a doctor with any disease
   - Flue
   - Covid-19
   - Strep throat
   - Cold
   - Other
8. Have you experienced one or more of the following symptoms in the last 24 hours?
   - My general feeling is good and I have no symptoms
   - Heat measured above 37.5
   - Cough
   - Sore throat
   - Runny nose
   - Headache
   - Shortness of breath
   - Muscle aches

- Weakness / fatigue
- Diarrhea
- Nausea / vomiting
- Chills
- Confusion
- Loss of sense of taste / smell
- Another symptom

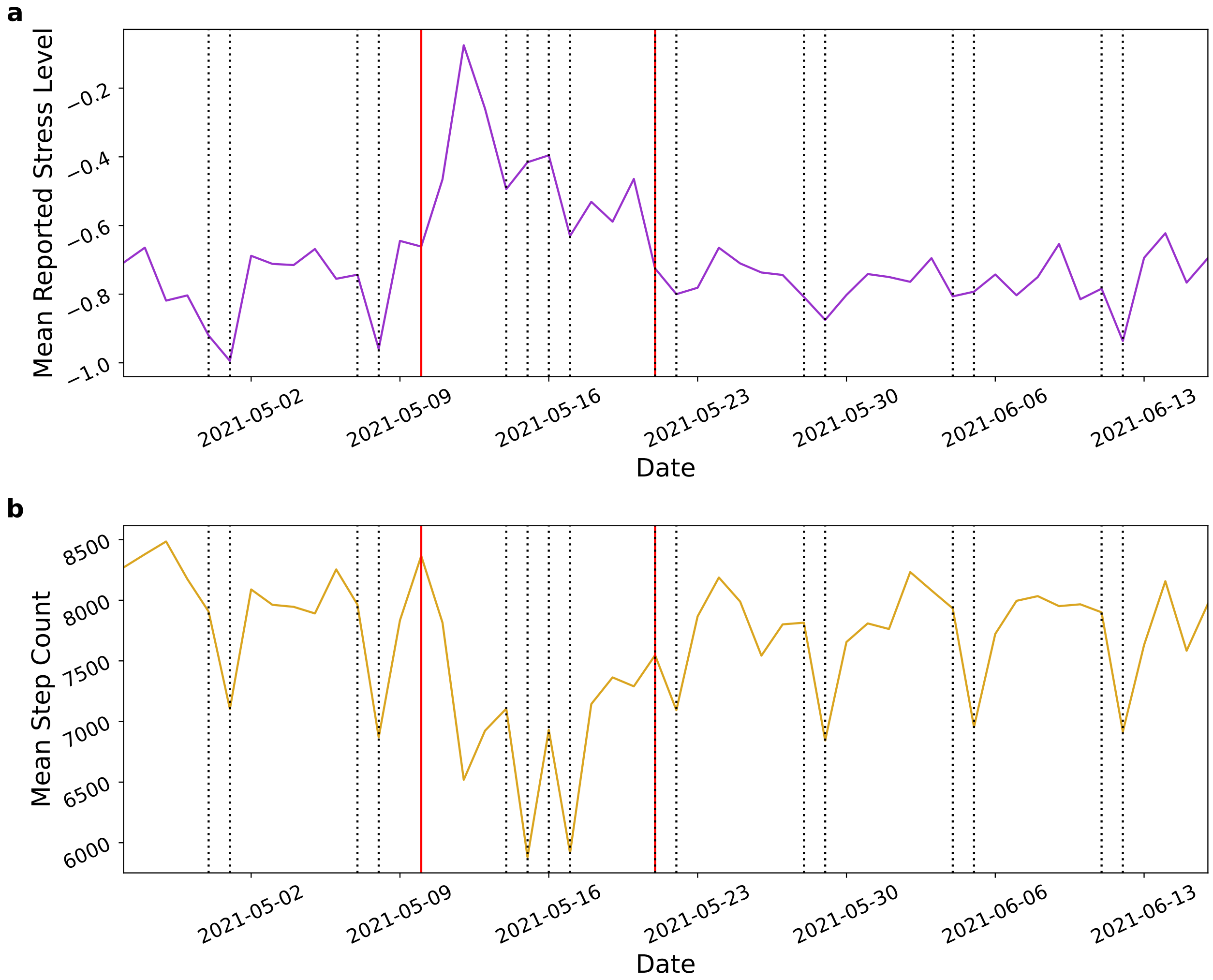

Supplementary Figure 2. Mean daily values for: (a) reported stress level and (b) step count. The x-axis represents time and the y-axis represents the mean daily value. The red vertical lines represent the beginning and the end of the war period, and the black vertical dashed lines indicate free days. Examination of the figure suggests the existence of a weekly rhythm, where for example, free days (weekends and national holidays) exhibit lower mean daily values than work days across both indicators.

Supplementary Notes 1

In Israel, when incoming missiles are spotted, air-raid sirens are turned on and civilians are instructed to head to shelters. The effective time to reach a shelter once an air-raid siren is turned on ranges from 15 to 180 seconds, depending on the area's distance from the closest missiles launch area. Notably, missiles against Israel can be launched from various countries, including Gaza in the south and Lebanon and Syria in the north.

The rationale behind our selection of the three exposure groups was mainly guided by the effective time to reach a shelter once an air-raid siren was turned on. However, as can be seen from Supplementary Figure 3, the effective time to reach a shelter alone is insufficient to define one's exposure group. That is, participants who live far away from Gaza and were not at risk to be hit by missiles during the current war cycle (for example, participants living in the north of Israel) are also associated with a short (below 90 seconds) effective time to reach a shelter. Therefore, instead of relying purely on the effective time to reach a shelter, we used the distance from Gaza. Namely, the lower threshold (60km) was chosen since it is able to capture the short effective time to reach a shelter for areas close to Gaza, and the upper threshold (110km) was chosen based on the maximum range of missiles fired from Gaza during this war cycle.

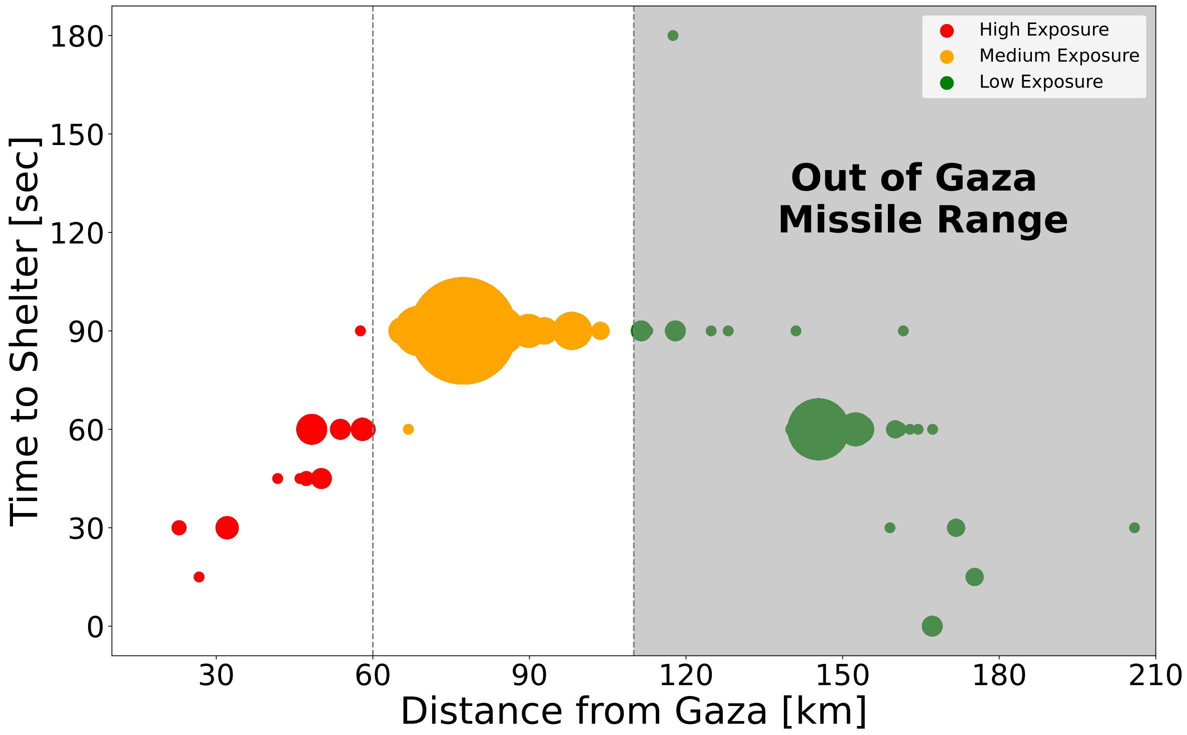

Supplementary Figure 3. Classification into exposure groups. The y-axis represents the effective time to reach a shelter and the x-axis represents the distance from Gaza. Each circle represents a set of participants sharing the same effective time to reach a shelter and distance from Gaza, where the area of the point is proportional to the size of the set. The grey area represents areas out of Gaza missile range during this war cycle.

		Population	Number of Participants	Baseline period (B)	War period (W)	First "back to routine" period (R1)	Second "back to routine" period (R2)
Mental	Screen-On Time [hour]	All	395	4.69±2.2	5.33±2.47	4.76±2.18	4.72±2.24
		Low Exposure	79	4.66±2.33	5.07±2.34	4.76±2.29	4.64±2.37
		Medium Exposure	290	4.67±2.15	5.32±2.44	4.75±2.14	4.72±2.17
		High Exposure	26	4.89±2.35	6.21±3.05	4.82±2.44	5.02±2.6
	Reported Mood Level	All	296	0.82±0.63	0.49±0.66	0.75±0.62	0.76±0.63
		Low Exposure	52	0.91±0.56	0.75±0.58	0.79±0.59	0.88±0.58
		Medium Exposure	221	0.79±0.65	0.45±0.66	0.73±0.62	0.72±0.63
		High Exposure	23	0.95±0.54	0.24±0.63	0.86±0.72	0.85±0.72
	Reported Stress Level	All	297	-0.75±0.62	-0.41±0.69	-0.73±0.61	-0.71±0.61
		Low Exposure	52	-0.85±0.54	-0.7±0.56	-0.8±0.54	-0.85±0.52
		Medium Exposure	222	-0.7±0.64	-0.37±0.71	-0.69±0.62	-0.66±0.62
		High Exposure	23	-0.96±0.54	-0.17±0.65	-0.9±0.67	-0.85±0.63
	Reported Number of Encounters	All	294	10.57±11.62	8.93±8.9	11.03±10.7	12.56±13.32
		Low Exposure	51	11.98±17.75	10.25±12.22	11.18±13.78	13.3±18.56
		Medium Exposure	220	10.23±10.13	8.87±8.29	10.99±10.11	12.5±12.17
		High Exposure	23	10.76±7.44	6.62±4.69	11.1±8.68	11.45±10.2
Energy	Step Count	All	499	7800.43±3936.45	7063.55±3725.11	7688.24±3890.37	7674.0±4003.71
		Low Exposure	121	7864.28±3767.93	7472.67±3862.79	7831.48±3871.38	8047.02±4054.18
		Medium Exposure	338	7683.57±3996.75	6870.39±3606.33	7584.56±3877.98	7482.25±3904.94
		High Exposure	40	8594.7±3920.91	7458.15±4230.35	8131.11±4103.4	8165.89±4620.76
	Average Heart Rate [bpm]	All	462	74.55±8.34	73.66±8.33	74.19±8.33	74.32±8.25
		Low Exposure	114	75.06±8.14	74.53±7.53	75.03±8.06	74.8±7.71
		Medium Exposure	312	74.51±8.46	73.45±8.53	73.97±8.42	74.26±8.49
		High Exposure	36	73.35±7.93	72.68±8.95	73.51±8.51	73.34±7.94
	% Time Still	All	402	87.27±7.09	88.66±7.18	87.08±7.21	86.86±6.79
		Low Exposure	80	87.13±7.58	88.46±6.54	87.57±6.88	87.32±6.26
		Medium Exposure	296	87.33±7.12	88.64±7.5	86.96±7.45	86.75±7.03
		High Exposure	26	87.02±5.21	89.53±5.1	86.92±5.2	86.7±5.56
	Reported Sport Time [min]	All	293	40.35±35.4	32.66±29.98	38.63±33.03	37.44±33.4
		Low Exposure	50	39.43±37.31	34.1±36.47	36.58±36.1	33.46±30.73
		Medium Exposure	220	40.33±31.57	33.58±28.87	39.49±31.02	37.91±31.49
		High Exposure	23	42.49±60.05	20.67±22.52	34.86±44.34	41.62±52.71
Sleep	Awake Time [sec]	All	316	768.61±670.86	987.7±677.99	774.6±657.41	758.46±617.42
		Low Exposure	69	824.27±774.13	1006.86±795.06	908.82±887.07	886.93±787.9
		Medium Exposure	226	759.16±655.29	988.16±658.06	744.87±590.39	719.45±553.28
		High Exposure	21	687.33±448.8	919.77±461.4	653.47±350.3	756.23±612.64
	Sleep Start Hour	All	316	23.77±1.7	23.91±1.4	23.82±1.48	23.78±1.56
		Low Exposure	69	23.6±2.52	23.69±1.5	23.62±1.87	23.58±2.56
		Medium Exposure	226	23.83±1.42	23.96±1.4	23.87±1.38	23.85±1.18
		High Exposure	21	23.64±1.01	24.1±1.06	23.96±0.94	23.69±0.64
	Reported Sleep Duration [hour]	All	297	6.43±0.87	6.27±0.96	6.41±0.87	6.41±0.87
		Low Exposure	52	6.44±0.84	6.48±0.88	6.41±0.78	6.36±0.89
		Medium Exposure	222	6.42±0.86	6.24±0.95	6.4±0.88	6.42±0.86
		High Exposure	23	6.57±1.05	6.11±1.13	6.49±0.89	6.36±0.89
	Reported Sleep Quality	All	297	0.49±0.62	0.31±0.68	0.5±0.64	0.51±0.65
		Low Exposure	52	0.58±0.57	0.5±0.54	0.6±0.57	0.63±0.61
		Medium Exposure	222	0.46±0.64	0.3±0.71	0.48±0.66	0.5±0.66
		High Exposure	23	0.52±0.46	-0.06±0.49	0.47±0.63	0.39±0.61

Supplementary Table 1. Descriptive statistics of the Mixed ANOVA tests. Number of participants, mean and standard error for each combination of well-being indicator, time period and exposure group. Each row represents a combination of a single well-being indicator and a single exposure group (or all groups together). Columns represent the number of participants considered in the analysis and the mean and standard error for each time period.

Supplementary Notes 2

This return to baseline values remained stable also in the succeeding two weeks period (R2). This remarkable recovery is also evidenced by the data presented in Supplementary Figure 4, showing that the changes between the baseline period (B) and the first "back to routine" period (R1) are distributed roughly normally around 0, with a relatively small standard deviation, and seemingly symmetric tails.

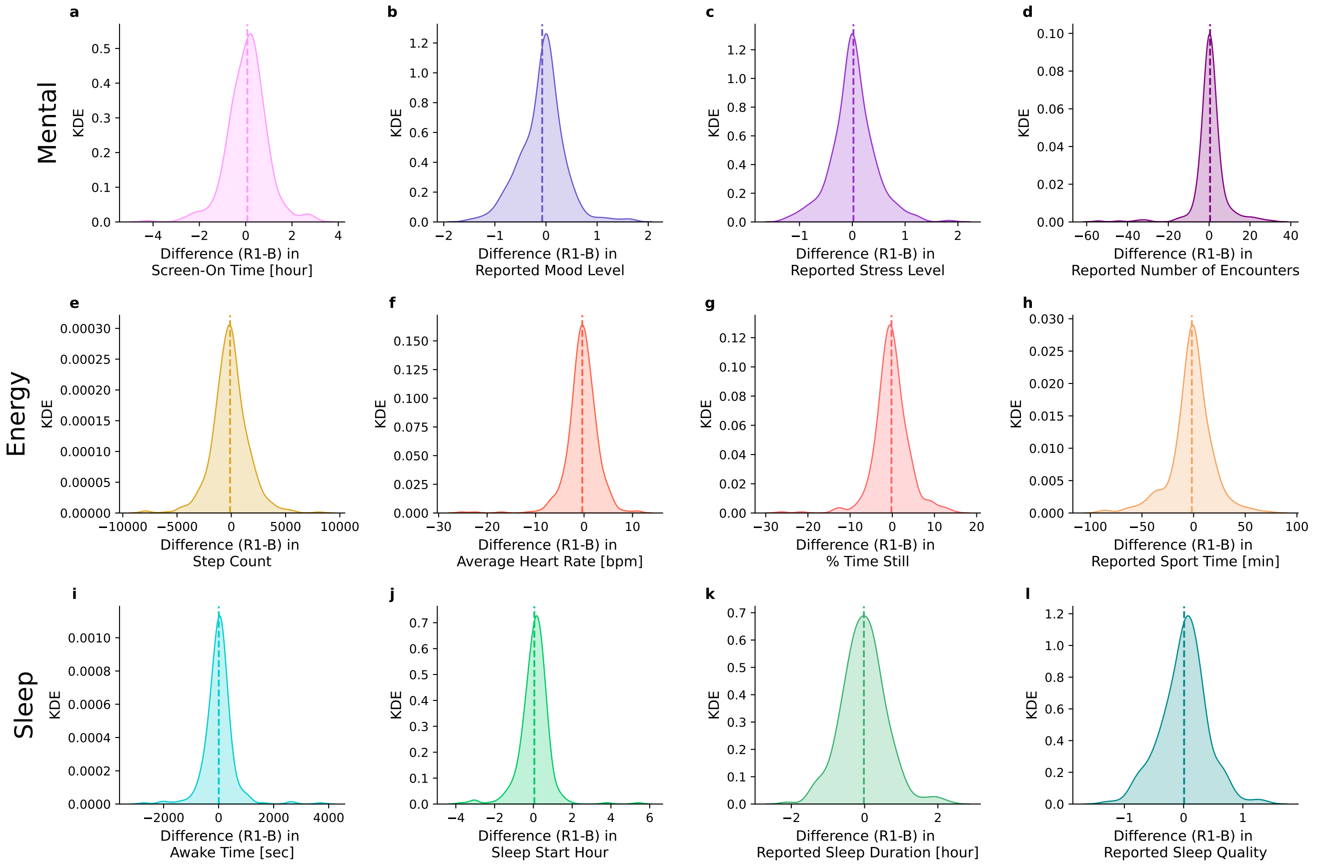

Supplementary Figure 4. Back to routine analysis - distribution of the difference between the first "back to routine" period (R1) and the baseline period (B) for various well-being indicators: (a) Screen time in hours (b) Reported mood level, (c) Reported stress level, (d) Reported number of encounters, (e) Step count, (f) Average heart rate in beats per minute, (g) Percentage of time still (h) Reported sport time in minutes, (i) Awake time during night sleep in seconds, (j) Sleep start hour (k) Reported sleep duration in hours, and (l) Reported sleep quality. The x-axis represents the mean difference value. The y-axis represents the kernel density estimator (KDE).

Supplementary Notes 3

Supplementary Figure 5 shows an example of a participant who does not seem to recover from the war effects. As can be seen from the figure, the participant presents roughly the same changes as the general population during the war period, but does not show a return to baseline values after the war in most of the examined well-being indicators. Specifically, after the war, the reported stress level continued to rise and the reported mood level, step count, reported sport time, reported sleep duration and reported sleep quality continued to drop. In contrast, number of encounters, average heart rate and awake time do seem to return to baseline values after the war.

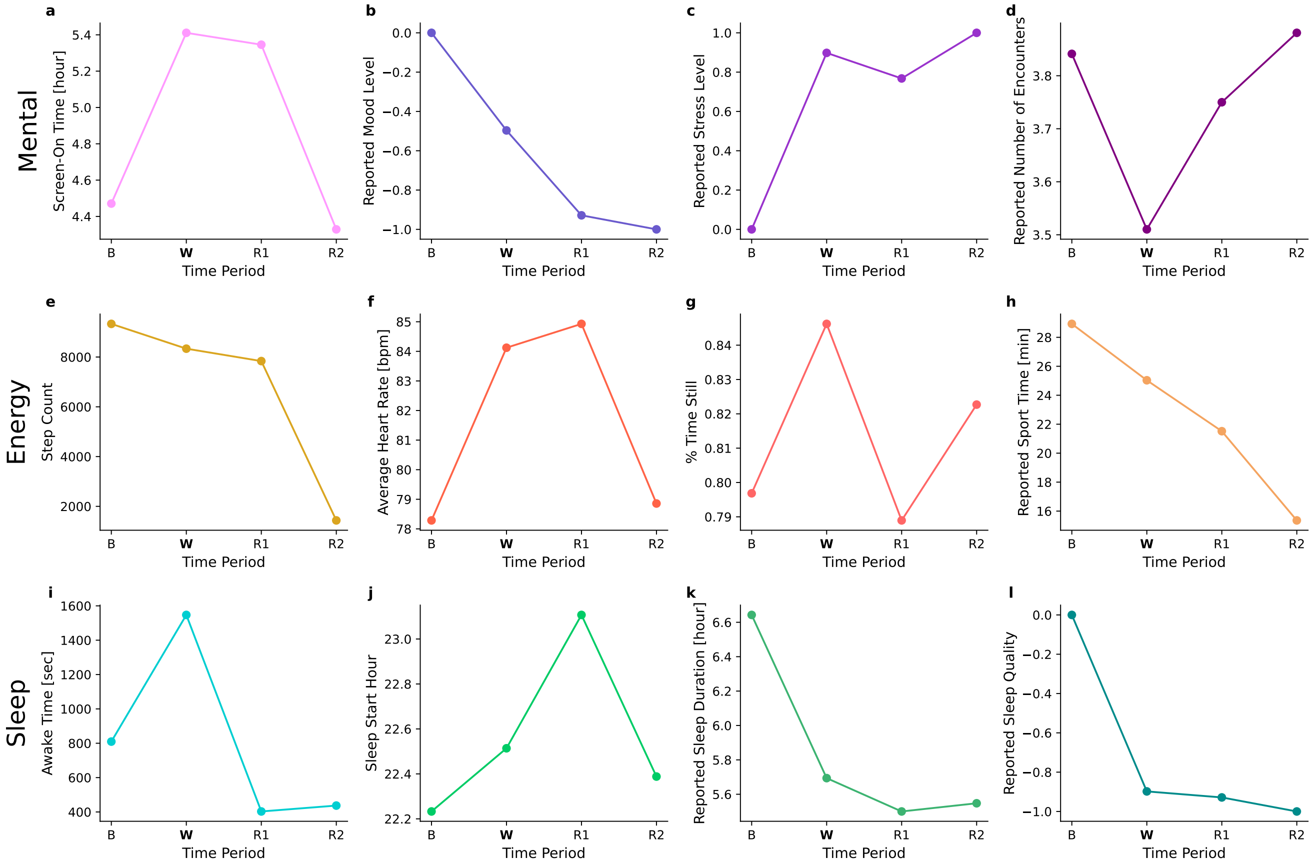

Supplementary Figure 5. An example of a participant who does not seem to recover from the war effects in most of the examined well-being indicators: (a) Screen-on time in hours (b) Reported mood level, (c) Reported stress level, (d) Reported number of encounters, (e) Step count, (f) Average heart rate in beats per minute, (g) Percentage of time still (h) Reported sport time in minutes, (i) Awake time during night sleep in seconds, (j) Sleep start hour (k) Reported sleep duration in hours, and (l) Reported sleep quality. The x-axis represents four time periods: baseline period (B), war period (W), first "back to routine" period (R1) and second "back to routine" period (R2). The y-axis represents the mean value for the examined well-being indicator.

		Exposure Group	Age Group	Gender	Income Level	Baseline Level
Mental	Screen-On Time [hour]	**0.003****	0.617	**0.022***	0.826	0.066
	Reported Mood Level	**0.003****	0.334	0.201	0.906	**0.00*****
	Reported Stress Level	**0.002****	0.639	**0.003****	0.852	**0.00*****
	Reported Number of Encounters	0.293	0.304	0.214	0.143	**0.00*****
Energy	Step Count	0.646	0.061	0.56	0.058	**0.00*****
	Average Heart Rate [bpm]	0.589	**0.036***	0.745	0.74	0.44
	% Time Still	0.276	0.401	0.453	0.481	**0.002****
	Reported Sport Time [min]	**0.002****	0.827	0.524	0.305	**0.00*****
Sleep	Awake Time [sec]	0.765	0.271	0.439	0.269	**0.00*****
	Sleep Start Hour	0.075	0.747	0.928	0.82	0.233
	Reported Sleep Duration [hour]	0.796	0.481	0.344	0.21	**0.001****
	Reported Sleep Quality	**0.001*****	0.209	0.382	0.898	**0.0*****

***p<0.001, **p<0.01**, p<0.05*

Supplementary Table 2. Post-hoc ANOVA tests. Each row represents ANOVA test for a single well-being indicator. Each column represents a single factor, and each entry represents the p-value of the corresponding effect. Statistically significant effects are marked with asterisks. For each test, the dependent variable was defined as the difference between the corresponding indicator values during the war and the baseline periods. For the independent variables, we considered exposure group, age group, gender, income level and baseline level. Each row represents a single indicator (i.e., a single test). Each ANOVA test was performed over the subset of participants who had at least one value in free days and at least one value in work days for the corresponding indicator, in both the war and the baseline periods.

		All Participants	Exposure Group				Age Group				Gender				Income Level						Baseline Level			
			Medium		High		Older		Younger		Women		Men		Lower than Median		Median		Higher than Median		Lower than Median		Higher than Median	
		N	N	Mean Difference	N	Mean Difference	N	Mean Difference	N	Mean Difference	N	Mean Difference	N	Mean Difference	N	Mean Difference	N	Mean Difference	N	Mean Difference	N	Mean Difference	N	Mean Difference
Mental	Screen-On Time [min]	316	289	37.74±65.12	27	76.49±68.12	178	40.44±70.11	138	41.84±60.95	184	49.32±68.99	132	29.53±60.4	98	43.55±72.47	63	36.4±64.13	155	41.36±63.08	157	32.43±54.29	159	49.57±75.31
	Reported Mood Level	295	267	-0.36±0.56	28	-0.7±0.48	178	-0.4±0.55	117	-0.37±0.58	179	-0.41±0.61	116	-0.36±0.48	92	-0.37±0.54	62	-0.35±0.51	141	-0.41±0.6	150	-0.22±0.46	145	-0.56±0.6
	Reported Stress Level	296	268	0.37±0.57	28	0.75±0.5	179	0.4±0.57	117	0.41±0.57	180	0.47±0.6	116	0.3±0.5	93	0.39±0.53	62	0.43±0.54	141	0.41±0.61	143	0.53±0.62	153	0.29±0.5
	Reported Number of Encounters	293	265	-1.28±8.8	28	-3.5±6.04	177	-0.98±6.5	116	-2.27±11.04	177	-1.24±8.58	116	-1.87±8.65	90	-2.37±10.64	62	-1.41±7.61	141	-0.96±7.49	139	0.74±3.71	154	-3.5±10.95
Energy	Step Count	386	342	-858.48±1950	44	-1131±2092	182	-577.65±1708	204	-1167.8±2136	207	-915.9±2028	179	-859.12±1896	100	-1141±1954	76	-769.34±2003	210	-813.32±1957	192	-114.82±1474	194	-1656.3±2091
	Average Heart Rate [bpm]	363	321	-1.02±2.97	42	-0.87±3.05	172	-0.66±2.53	191	-1.32±3.3	196	-1.07±2.71	167	-0.93±3.26	94	-0.98±2.22	75	-1.24±3.57	194	-0.92±3.05	182	-0.85±2.43	181	-1.16±3.43
	% Time Still	322	295	1.07±6.85	27	2.66±3.97	181	1.36±4.74	141	1.0±8.54	188	1.08±4.68	134	1.37±8.74	99	1.81±4.59	65	0.51±4.99	158	1.11±8.19	164	2.31±8.38	158	0.05±3.92
	Reported Sport Time [min]	292	264	-7.53±23.26	28	-20.48±38.11	176	-8.09±26.48	116	-9.81±23.37	176	-7.22±18.24	116	-11.13±33.16	89	-4.51±19.19	62	-10.72±32.72	141	-10.61±24.74	146	-1.46±11.24	146	-16.08±32.35
Sleep	Awake Time [sec]	256	232	240.71±582.35	24	298.35±359.06	150	169.13±613.09	106	355.05±470.31	148	269.6±558.31	108	213.92±574.89	78	143.07±698.49	49	315.57±567.79	129	282.03±460.17	134	407.71±418.82	122	68.62±647.35
	Sleep Start Hour	256	232	0.16±0.74	24	0.45±0.59	150	0.19±0.73	106	0.18±0.74	148	0.18±0.79	108	0.19±0.66	78	0.13±0.94	49	0.23±0.62	129	0.19±0.63	125	0.24±0.68	131	0.13±0.78
	Reported Sleep Duration [hour]	296	268	-0.24±0.81	28	-0.29±0.81	179	-0.23±0.75	117	-0.28±0.9	180	-0.3±0.89	116	-0.16±0.66	93	-0.35±0.91	62	-0.3±0.83	141	-0.15±0.72	148	-0.08±0.8	148	-0.41±0.79
	Reported Sleep Quality	296	268	-0.2±0.5	28	-0.56±0.44	179	-0.2±0.51	117	-0.29±0.5	180	-0.26±0.52	116	-0.2±0.5	93	-0.25±0.54	62	-0.2±0.47	141	-0.25±0.51	153	-0.13±0.46	143	-0.36±0.53

Supplementary Table 3. Descriptive statistics of the post-hoc ANOVA tests. Number of participants, mean and standard error are presented for each combination of well-being indicator and factor level. The table presents detailed information for each ANOVA test about the number of participants and the mean and the standard error for each combination of well-being indicator and factor level.

Supplementary Notes 4

Supplementary Figure 6 presents the Pearson correlation values between each pair of the 12 examined well-being indicators. For this purpose, we considered all participants that had at least one value in free days and at least value in work days for the corresponding indicator during the baseline period. Then, Pearson correlation between two indicators was calculated for the two corresponding vectors of values (corresponding entries in these two vectors represent a single individual which had a valid value for both indicators). As can be seen from the figure, several well-being indicators are highly correlated. For example, the Pearson correlation between reported mood level and reported stress level is -0.8 ($p<0.001$) and the Pearson correlation between reported sport time and smartwatch's steps count is 0.37 ($p<0.001$).

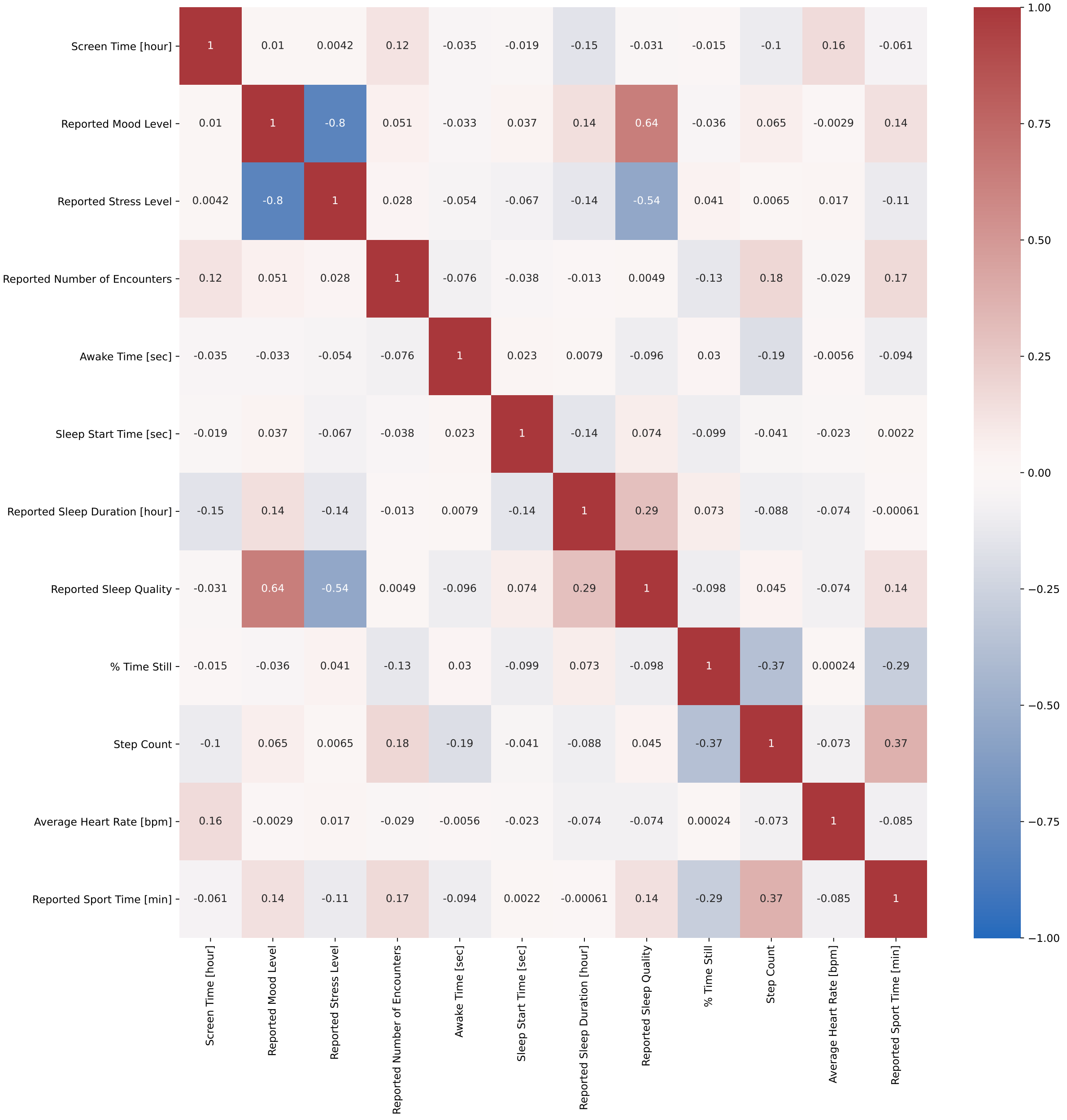

Supplementary Figure 6. Correlation matrix of well-being indicators. Each cell represents the Pearson correlation value between a pair of well-being indicators.
