# Supplementary material for: Real-time sensing of war’s effects on wellbeing with smartphones and smartwatches
Source: Commun Med (Lond). 2023 Apr 17;3:55. doi: 10.1038/s43856-023-00284-y (PMC10109229; doi:10.1038/s43856-023-00284-y)
Supplement: Supplementary file 2 — Reporting Summary [file 43856_2023_284_MOESM2_ESM.pdf]

nature portfolio

Corresponding author(s): Prof. Erez Shmueli

Last updated by author(s): Feb 17, 2023

Reporting Summary

Nature Portfolio wishes to improve the reproducibility of the work that we publish. This form provides structure for consistency and transparency in reporting. For further information on Nature Portfolio policies, see our Editorial Policies and the Editorial Policy Checklist.

Statistics

For all statistical analyses, confirm that the following items are present in the figure legend, table legend, main text, or Methods section.

n/a	Confirmed	
☐	☒	The exact sample size (*n*) for each experimental group/condition, given as a discrete number and unit of measurement
☐	☒	A statement on whether measurements were taken from distinct samples or whether the same sample was measured repeatedly
☐	☒	The statistical test(s) used AND whether they are one- or two-sided<br>*Only common tests should be described solely by name; describe more complex techniques in the Methods section.*
☒	☐	A description of all covariates tested
☒	☐	A description of any assumptions or corrections, such as tests of normality and adjustment for multiple comparisons
☐	☒	A full description of the statistical parameters including central tendency (e.g. means) or other basic estimates (e.g. regression coefficient) AND variation (e.g. standard deviation) or associated estimates of uncertainty (e.g. confidence intervals)
☐	☒	For null hypothesis testing, the test statistic (e.g. *F*, *t*, *r*) with confidence intervals, effect sizes, degrees of freedom and *P* value noted<br>*Give P values as exact values whenever suitable.*
☒	☐	For Bayesian analysis, information on the choice of priors and Markov chain Monte Carlo settings
☒	☐	For hierarchical and complex designs, identification of the appropriate level for tests and full reporting of outcomes
☐	☒	Estimates of effect sizes (e.g. Cohen's *d*, Pearson's *r*), indicating how they were calculated

Our web collection on statistics for biologists contains articles on many of the points above.

Software and code

Policy information about availability of computer code

Data collection	Data were collected using custom code, programmed using Python 3.7 environment. The storage server runs a CentOS operating system.
Data analysis	Data were analyzed using Python 3.7 programming environment and IBM SPSS Statistics version 27.

For manuscripts utilizing custom algorithms or software that are central to the research but not yet described in published literature, software must be made available to editors and reviewers. We strongly encourage code deposition in a community repository (e.g. GitHub). See the Nature Portfolio guidelines for submitting code & software for further information.

Data

Policy information about availability of data

All manuscripts must include a data availability statement. This statement should provide the following information, where applicable:

- Accession codes, unique identifiers, or web links for publicly available datasets
- A description of any restrictions on data availability
- For clinical datasets or third party data, please ensure that the statement adheres to our policy

Researchers interested in obtaining an aggregated version of the data sufficient to reproduce the results reported in this paper should contact the corresponding author.

Human research participants

Policy information about studies involving human research participants and Sex and Gender in Research.

Reporting on sex and gender	Gender was determined based one enrollment questionnaire filled with survey company representative, after signing a digital consent form. Out of 954 sample size, 42.45% (405) were men and 57.55% (549) were women.<br>In the context of gender, we investigated the characteristics of participants who reported a higher rise in their stress level during the war compared to the baseline period. We divided the set of participants into two groups based on their ranking above or below the median in terms of reported rise in the stress level. Then, we looked at the difference between the two groups in terms of gender.
Population characteristics	See behavioral & social sciences study design section
Recruitment	In order to recruit subjects and keep them engaged throughout the PerMed study, we hired a professional survey company. The survey company used advertisements on social media for recruitment of individuals from the general population.The survey company was responsible to guarantee that participants met the study's requirements, including their willingness to fill an app questionnaire three times a week and wear a smartwatch during the entire study. Eligible participants received a detailed explanation about the study, after which they were requested to sign a digital consent form. Then, participants were asked to fill a one-time enrollment questionnaire and to install two apps on their smartphones: the Garmin Connect app which was used to collect data from their smartwatch, and the dedicated PerMed app which we developed to collect smartphones sensors and GPS-based location and to allow participants to fill the daily questionnaires.
Ethics oversight	The study was approved on August 8, 2021, by Tel-Aviv University's Institutional Review Board (IRB) and was conducted under strict protocol guidelines.

Note that full information on the approval of the study protocol must also be provided in the manuscript.

Field-specific reporting

Please select the one below that is the best fit for your research. If you are not sure, read the appropriate sections before making your selection.

☐ Life sciences ☒ Behavioural & social sciences ☐ Ecological, evolutionary & environmental sciences

For a reference copy of the document with all sections, see nature.com/documents/nr-reporting-summary-flat.pdf

Behavioural & social sciences study design

All studies must disclose on these points even when the disclosure is negative.

Study description	In this study we will analyze data that was already collected and will be collected as part of the PerMed study. Participants in the PerMed study are recruited for a period of two years, during which they are equipped with a Garmin Vivosmart 4 smartwatches and are asked to wear them as much as they could. In addition, participants install two applications on their mobile phones: an application that passively collects data from the smartwatch and a dedicated mobile application which allows participants to fill a daily questionnaire and that passively collect mobile sensor data.
Research sample	The study included a cohort of 954 participants above the age of 40 with a median age of 59. Of the 954 participants, 549 (57.55%) were women and 405 (42.45%) were men. The reported income of 475 (49.79%) participants was above the median income level, while that of 185 (19.39%) was in the median income range, and that of 258 (27.04%) was below the median; 36 did not answer the relevant question in the enrollment questionnaire. In terms of exposure to missile attacks, 68 (7.13%) participants lived in high risk areas, 704 (73.79%) in medium risk areas and 182 (19.08%) were not exposed to missile attacks at all.
Sampling strategy	The sampling procedure was convenience sampling. Participants recruited through advertisements in social media, online banners, and word-of-mouth.
Data collection	Participants were equipped with Garmin Vivosmart 4 smart fitness trackers. Among other features, the smartwatch provides daily measures such as steps count, average heart rate, sleep start hour, and percentage of awake time during night. All participants asked to complete daily self-reported questionnaire in a dedicated application (the PerMed mobile application). Among other questions, the daily questionnaire includes questions about mood, stress, sleep quality, sleep duration, sport time and number of social encounters. The PerMed app also collected GPS location and daily aggregated smartphone sensors including screen-on time and the percentage of time still (according to Google Activity Recognition).
Timing	We define four time periods:(1) Baseline period (B) - the two weeks before the war, i.e., April 26--May 9, 2021;(2) War period (W) - May 10--20, 2021;(3) First ``back to routine'' period (R1) - the first and second weeks after the war, i.e., May 21--June 3, 2021; and(4) Second ``back to routine'' period (R2) - the third and fourth weeks after the war, i.e., June 4--17, 2021.

Data exclusions	We included in the study participants of the PerMed study who were above the age of 40 and were active in the PerMed study throughout the evaluation period (i.e., they joined the PerMed study before April 26, 2021, and remained in the study at least until June 17, 2021).
Non-participation	No participants dropped out/declined participation.
Randomization	N/A

Reporting for specific materials, systems and methods

We require information from authors about some types of materials, experimental systems and methods used in many studies. Here, indicate whether each material, system or method listed is relevant to your study. If you are not sure if a list item applies to your research, read the appropriate section before selecting a response.

Materials & experimental systems

n/a	Involved in the study	
☒	☐	Antibodies
☒	☐	Eukaryotic cell lines
☒	☐	Palaeontology and archaeology
☒	☐	Animals and other organisms
☒	☐	Clinical data
☒	☐	Dual use research of concern

Methods

n/a	Involved in the study	
☒	☐	ChIP-seq
☒	☐	Flow cytometry
☒	☐	MRI-based neuroimaging
